# Supplementary material for: High-Affinity Plasma Membrane Ca2+ Channel Cch1 Modulates Adaptation to Sodium Dodecyl Sulfate-Triggered Rise in Cytosolic Ca2+ Concentration in Ogataea parapolymorpha
Source: Int J Mol Sci. 2024 Oct 25;25(21):11450. doi: 10.3390/ijms252111450 (PMC11546840; doi:10.3390/ijms252111450)
Supplement: Supplementary file 1 [file ijms-25-11450-s001.zip › ijms-3225564-supplementary.pdf]

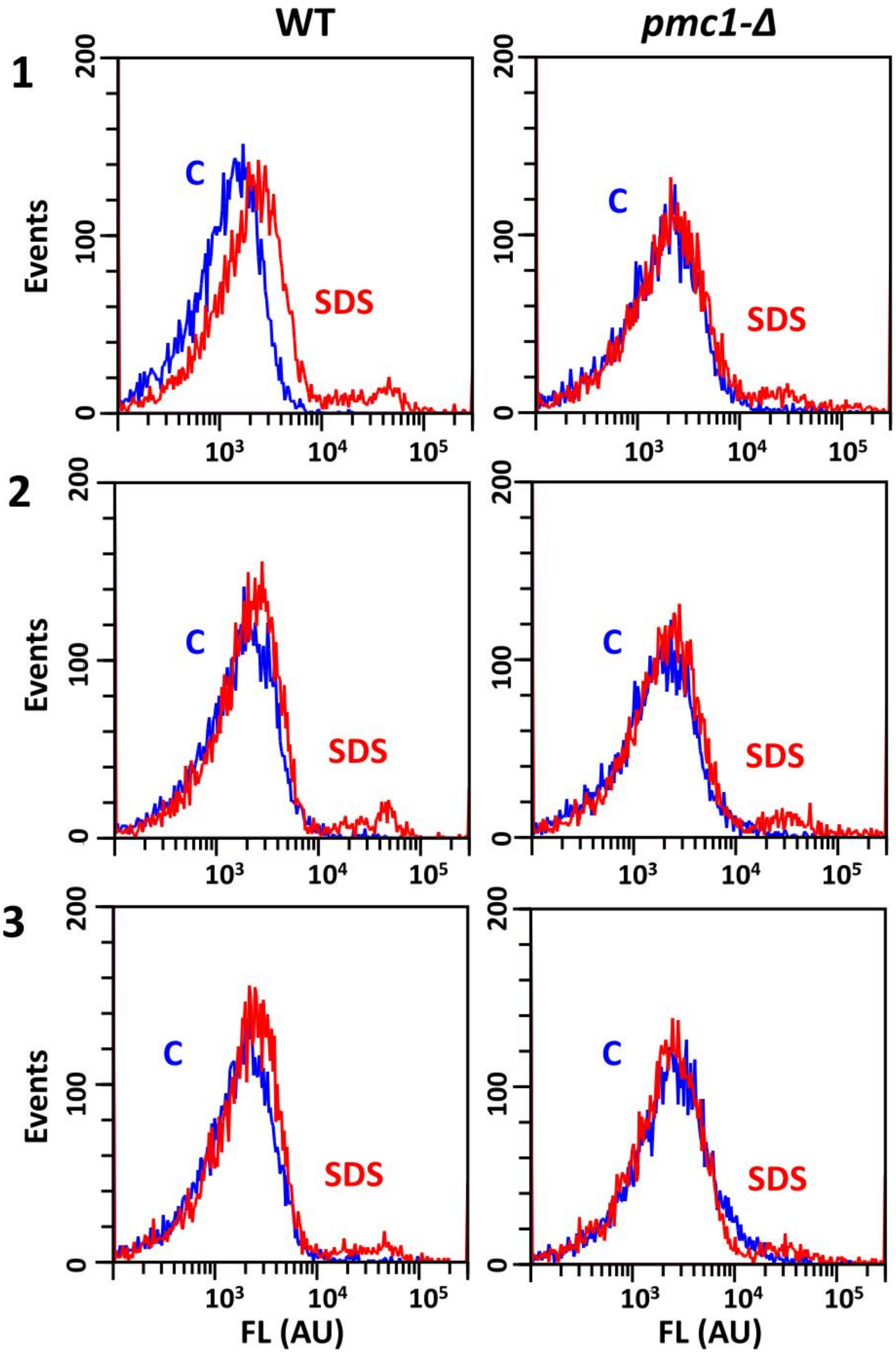

Figure S1. Distribution of the fluorescence (FL) of DL5-LC (WT) and DL5-pmc1-LC (*pmc1-Δ*) cells after PI staining . Red line “SDS”, cells after 1 h incubation with 0.01% SDS; blue line “C”, untreated control cells. The data from different replicates are marked as 1, 2, and 3.
